# Supplementary material for: Remote assessment in adults with Autism or ADHD: A service user satisfaction survey
Source: PLoS One. 2021 Mar 25;16(3):e0249237. doi: 10.1371/journal.pone.0249237 (PMC7993762; doi:10.1371/journal.pone.0249237)
Supplement: S1 Appendix — (PDF) [file pone.0249237.s001.pdf]

NHS
South West
Yorkshire Partnership
NHS Foundation Trust

Service User Feedback for ADHD and Autism Assessments during Covid 19

Which service have you used?
☐ADHD
☐Autism

What was your assessment for?
☐Diagnostic Assessment
☐Medication Review
☐Psychological Intervention

---

1. **Were you pleased to receive a telephone / video appointment during the coronavirus restrictions?**
   ☐Yes completely
   ☐Yes to some extent
   ☐No
   ☐Don’t know

2. **Would you have preferred a face to face appointment?**
   ☐Yes
   ☐No
   ☐Don't know

3. **Did the appointment letter explain what would happen during the assessment?**
   ☐Yes, completely
   ☐Yes, to some extent
   ☐No
   ☐Don’t know

4. **Did you receive support during your appointment?**
   ☐Yes, from a family member
   ☐Yes, from my partner
   ☐Yes, from a carer
   ☐Yes, from someone else
   ☐No

5. **How did we contact you? (tick all that apply)**
   ☐Telephone Consultation
   ☐Video Consultation

6. **If you did not have a video call what was the reason?**
   ☐Not given the option
   ☐Did not have the appropriate equipment
   ☐Did not have internet connection
   ☐Other (please provide detail)

To protect the environment and save money this letter is printed on recycled and unbleached paper.

7. **Were you contacted at the agreed time?**
   ☐Yes
   ☐No
   ☐Don't know

8. **How would you rate the quality of the telephone / video call? Please circle.**
   (1 being poor and 5 being excellent)

   1 2 3 4 5

9. **How well do you think you were able to communicate over the telephone / video call?**
   ☐Very well
   ☐Well
   ☐Not very well
   ☐Don’t know

10. **Were you able to find somewhere appropriate to speak to your clinician during your telephone / video call?**
    ☐Yes
    ☐No
    ☐Don't know

11. **Did you have enough time to answer the questions?**
    ☐Yes completely
    ☐Yes to some extent
    ☐No
    ☐Don’t know

12. **Do you think you would have been able to explain yourself better if you had been seen face to face?**
    ☐Yes completely
    ☐Yes to some extent
    ☐No
    ☐Don’t know

13. **Do you feel the clinician completed a detailed initial assessment by telephone / video?**
    ☐Yes
    ☐No
    ☐Don't know

14. **Do you feel it was explained to you what would happen following your assessment?**
    ☐Yes
    ☐No
    ☐Don't know

15. **Do you think we should continue to offer telephone / video appointments after coronavirus restrictions are lifted?**
    ☐Yes
    ☐No
    ☐Don't know

16. **Would you tell your friends and family that this is a good service?**
    ☐Yes
    ☐Maybe
    ☐No
    ☐Don’t know

17. What was good about the service?

18. What could have been better?

In order to ensure that we provide the best service for **all** of our communities, and to ensure that we do not knowingly discriminate against any section of society, it is important for us to gather the following information. *You do not have to answer any of these questions, but we would be very grateful if you would.*

What is the first part of your postcode?

How would you describe yourself?

☐A service-user
☐A carer/ unpaid carer (relative/friend/neighbor etc.)
☐Member of a Voluntary or community group
☐A SWYT staff member
☐Other please state ………

How old are you:

What is your ethnicity?

What is your gender?

Are you happy for your comments to be made public? ☐Yes ☐No
